# Development and proof-of-concept evaluation for a low resource compatible Chikungunya virus diagnostic

**DOI:** 10.1371/journal.pntd.0012352

**Published:** 2025-09-12

**Authors:** Rickyle Balea, Alberto A. Amarilla, Jody Hobson-Peters, Joanne Macdonald, Andreas Suhrbier, Vasilli M. Kasimov, Daniel Watterson, Nina M. Pollak, David J. McMillan

**Affiliations:** 1 School of Science, Technology and Engineering, University of the Sunshine Coast, Sippy Downs, Queensland, Australia; 2 School of Chemistry and Molecular Biosciences, The University of Queensland, St. Lucia, Queensland, Australia; 3 Centre for Bioinnovation, University of the Sunshine Coast, Sippy Downs, Queensland, Australia; 4 BioCifer Pty Ltd, Buderim, Queensland, Australia; 5 QIMR Berghofer Medical Research Institute, Herston, Queensland, Australia; 6 Institute for Glycomics, Griffith University, Gold Coast, Queensland, Australia; Southern Medical University, CHINA

## Abstract

Chikungunya virus (CHIKV) is a positive sense RNA *Alphavirus* that continues to pose major public health threats throughout the world. CHIKV is primarily transmitted via the Aedes genus mosquito; however, has also exhibited transmission routes via blood transfusion and vertical transmission (mother to child). With only one approved vaccine thus far and no approved medicines or specific therapeutics, early detection is crucial in mitigating potential CHIKV outbreaks. Here, we designed and evaluated a sensitive and specific CHIKV diagnostic using reverse transcription-recombinase aided amplification (RT-RAA) coupled lateral flow strip detection (LFD) targeting a highly conserved region of the CHIKV E1 gene. Our results demonstrate that using our simple sample preparation reagent (TNA-Cifer-E), we can inactivate live CHIKV in two minutes at room temperature, whilst also sustaining viable viral RNA. Our specificity analysis demonstrates the Iso-CHIKV-Dx does not detect any closely related *Alphaviruses* nor any of the common co-circulating *Flaviviruses*. Proof-of-concept evaluation using urine spiked with CHIKV exhibited that in CHIKV infected urine samples, our Iso-CHIKV-Dx can detect as low as 570 copies/µL of CHIKV RNA in 30 minutes under isothermal conditions. Contrary to conventional RT-qPCR, our Iso-CHIKV-Dx does not require expensive machinery, advanced instrumentation or extensively trained personnel. Further performance comparisons also show that our Iso-CHIKV-Dx is four times faster than conventional RNA isolation and RT-qPCR. As such, pre-clinical, proof-of-concept evaluation demonstrates that our Iso-CHIKV-Dx has the potential to act as a robust, point of care CHIKV diagnostic that could prove to be highly beneficial in place of, or in the absence of conventional diagnostic approaches such as RT-qPCR.

## Author summary

There is currently a significant concern expressed by various global health organisations (WHO, CDC, EMA etc) that the next pandemic could be caused by an arbovirus. The emergence and/or re-emergence of arboviruses such as Chikungunya virus (CHIKV) are of particular concern due to driving factors such globalisation, vector compentency, increasing urbanisation and climate change. There is still a significant lack of approved medicines and specific therapeutics for CHIKV. Therefore, rapid diagnostics and early detection continue to serve as crucial measures in preventing and controlling major outbreaks. Typically, CHIKV outbreaks occur within remote, rural settings; areas often lacking in resources and extensively trained personnel. As such, in this study we developed and evaluated a potential point-of-care rapid CHIKV diagnostic that can be implemented without the need for advanced instrumentation or experienced personnel. This unique approach to arbovirus detection could prove to offer promising insights into novel innovations within the field of rapid diagnostics and preparedness for arbovirus outbreaks.

## Introduction

The emergence and re-emergence of pathogenic arboviruses over recent years has been a significant area of concern regarding global public health. As a result, in 2022, the ‘Global Arbovirus Initiative’ [1] was launched by the World Health Organisation (WHO). Among the various arboviruses listed, Chikungunya virus (CHIKV) was among the top three of the major arboviruses of concern. Chikungunya virus is a positive sense RNA *Alphavirus* belonging to the *Togaviridae* family [2]. Clinical manifestations of CHIKV infections include fever, aggressive arthralgia and inflammatory musculoskeletal disease resulting in debilitating pain and discomfort [3,4]. Whilst death is often rare with CHIKV infections, viremic pathogenesis has been identified in numerous cell types throughout the body; including dendritic cells, macrophages, synovial fibroblasts, endothelial cells, myocytes as well as osteoblasts [4–6]. Whilst CHIKV is primarily transmitted to humans via blood meal from an *Aedes aegpyti* or *Aedes albopictus* mosquito [7], several cases of vertical transmission (mother to child) and blood transfusion have been reported [8]. Although there are currently no specific therapeutics or medicines for CHIKV infections [9,10], in early 2024, the FDA in the United States formally approved the vaccine ‘IXCHIQ’ [10] for CHIKV.

CHIKV was first isolated in 1952 from the serum of a patient in Tanzania displaying arthrogenic like symptoms [3]. For several years, CHIKV exclusively circulated throughout northern Africa, with the exception of a few minor clusters in Thailand and the Philippines within 1950’s [11]. In 2005–06 however, a large outbreak occurred in the Reunion Islands [3] in which a third of the population (775, 000) were infected and 237 deaths had occurred [12]. Chikungunya virus has now spread rapidly throughout six continents and has been reported in more than 40 countries [13]. Due to its vast global prevalence and the significant lack of approved medicines and therapeutics, early detection and diagnosis remain crucially vital in mitigating potential CHIKV outbreaks. Currently, diagnostic techniques such as RNA isolation and RT-qPCR are regarded as the gold standard approach for CHIKV diagnostics [14–16]. However, factors such as the need for experienced laboratory personnel, intricate methodology, and requirement of advanced instrumentation (thermal cyclers), typically render RT-qPCR as unfavourable within low-resource locations. To that end, whilst CHIKV does exhibit a vast global distribution, the majority of outbreaks are consistently observed in rural, remote settings [17]. These settings are often observed to be lacking in resources, personnel and advanced instrumentation. Comparatively, rapid isothermal nucleic acid amplification tests (iNAATs) that operate without the need for these elements appear to be gaining significant traction [18].

Isothermal amplification techniques such as Reverse Transcription-Loop Mediated Amplification (RT-LAMP) [19] and Reverse Transcription-Recombinase Polymerase Amplification (RT-RPA) [20] have demonstrated highly promising results in the detection of several Arboviruses thus far [21,22]. However, they have not been assessed extensively within the scope of rapid, point-of-care (POC) CHIKV diagnostics. Recombinase Aided Amplification (RAA) [23] is an iNAAT similar to RPA [24] that has demonstrated promising results within the field of infectious disease diagnostics [25,26]. With the inclusion of a reverse transcriptase (RT) enzyme, RT-RAA demonstrated promising results in detecting arboviruses such as Zika virus (ZIKV) [27]. So far, RT-RAA has exhibited clinically rapid [25] and sensitive [28] attributes. Furthermore, implementation of RT-RAA within an isothermal diagnostic tool demonstrated promising pre-clinical efficacy for rapid detection of ZIKV in urine samples [27] when compared to RT-qPCR. Despite the ongoing efforts to develop medicines and vaccines for CHIKV the current threats toward global public health call for a more immediate measure for controlling and preventing major outbreaks. As such, early detection and rapid diagnosis remain crucially vital in mitigating CHIKV outbreaks.

Here we report the development and pre-clinical evaluation of a rapid, isothermal CHIKV diagnostic platform (Iso-CHIKV-Dx). Our diagnostic platform utilises a unique, low-resource compatible sample processing protocol, a rapid isothermal (RT-RAA) procedure and lateral flow strip detection (LFD) method that detects CHIKV infections in under 30 minutes. This rapid POC-like CHIKV diagnostic requires minimal laboratory experience nor the need for expensive instrumentation. As such, our Iso-CHIKV-Dx demonstrates promising detection and diagnostic capabilities for CHIKV infections, particularly within low-resource dependant settings.

## Methods and materials

### Plasmids and RNA template preparation

A plasmid (pBIC-A) containing regions of the CHIKV Envelope (E1) gene fragment (LC500222.1) was obtained from Bioneer Pacific Pty Ltd, Victoria, AUS). RNA transcripts were generated using the MEGAscript T7 transcription kit (Invitrogen by Thermo Fisher Scientific Australia Pty Ltd, Victoria, AUS) as previously described [29].

### RAA primer and probe design

**Fig 1:**
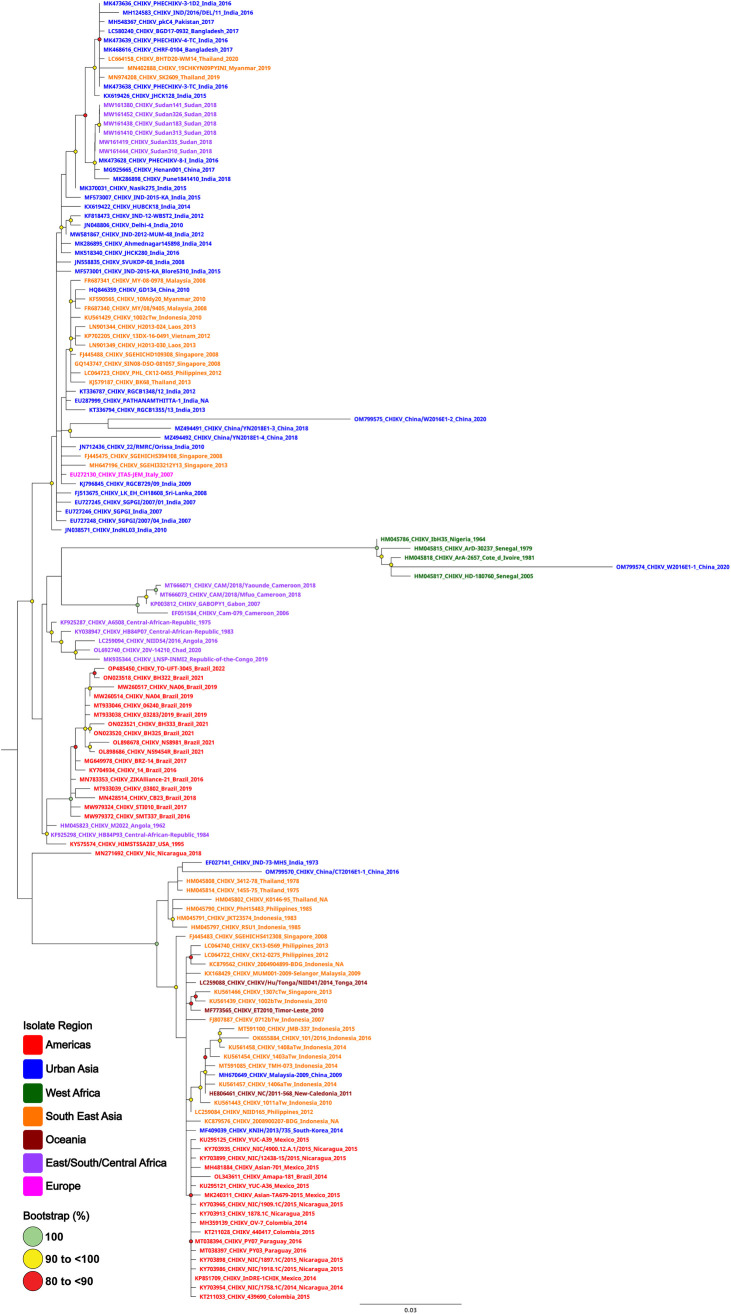
Phylogenetic tree of 141 CHIKV E1 gene sequences. CHIKV E1 sequences are named by their corresponding GenBank accession number/strain/country of origin, with text being coloured according to their continent of isolation. The midpoint-rooted maximum likelihood phylogenetic trees were constructed using IQTREE2, which automatically incorporates the most appropriate nucleotide substitution model (TN + F + G4). The scale bar indicates the number of nucleotide substitutions per site. Bootstrap values above 80% are displayed next to the node of each clade and are coloured according to the figure legend.

A total of 660 CHIKV E1 gene sequences representing the East Central and South African (ECSA), West African, Urban Asia, Southeast Asia, Oceania, Europe and the Americas lineages were obtained from the NCBI GenBank nucleotide database. All 660 E1 sequences were aligned using MAFFT (v7.49), as implemented in Geneious Prime (version 2023.0.4) [30], where a total of 141 unique E1 gene sequences were identified. All 141 unique sequences were realigned using MAFFT [31] and IDTREE2 [32] was used to construct a maximum likelihood (ML) phylogenetic tree (Fig 1) [32], utilising the in-built TEST function which incorporates the most appropriate nucleotide substitution model (TN + F + G4) and 10,000 bootstrap replicates. Primers and probes were then designed to target the most conserved region of E1 gene (~2000..2350) throughout the consensus sequence. Primer and probe design were further evaluated (primer dimers, secondary structures and GC content) using an oligoevaluator [33] and subsequently synthesised by Bioneer Pacific (Pty Ltd, Victoria, AUS) (Table 1).

**Table 1:** Primer and probe sequences for rapid Iso-CHIKV-Dx assay.

Oligonucleotide	Sequence
Forward Primer	GACGTCTATGCTAAYACHCAACTGGTACTGC
Reverse Primer	[5’ Biotin] CTCTTACCGGGTTTGTTGCTATTTGRCAGCC
Probe	[5’FAM] CAGGCACCATCTGGCTTYAAGTATTGGYTDA [Internal dS Spacer] AGAACGVSGGGCGTC [3’ C3 spacer]

## Iso-CHIKV-Dx assay

### Rapid sample processing

CHIKV ‘Mauritius strain’ culture (GenBank ID: EU404186) 2.5 x 10^7^ FFU/ml was mixed into urine samples at a ratio of 1:2 with TNA-Cifer Reagent E (BioCifer, Auchenflower, AUS) at room temperature for 2 minutes. Simulated urine samples were further diluted 1:10 in nuclease free H_2_0.

### RT-RAA amplification

Each RT-RAA assay was performed using an RAA kit (Jiangsu Qitian Gene Biotechnology Co. Ltd, Wuxi City China) with final reaction conditions of 1x RAA rehydration buffer, 1/5 RAA pellet, forward primer (420 nM), reverse primer (420 nM), probe (120 nM), Endonuclease IV (2 U; New England Biolabs, Victoria, AUS), Moloney Murine Leukemia virus reverse transcriptase enzyme (M-MLV, 60 U; Biocifer Pty Ltd, Auchenflower, AUS), magnesium acetate (MgOAc, 14mM) and 1 µL template in a final reaction volume of 10 µL. MgOAc was added to the cap of each 0.2 ml PCR tube. Once template had been added, tubes were spun down and incubated at 39°C for 20 minutes using a heating block.

### Lateral flow strip detection

HybriDetect lateral flow strips (LFS) (Milenia Biotec, Giessen, GER) were treated with 8 µL of 0.4% casein blocking buffer for pre-activation [34]. To each strip, 2 µL of amplicon was pipetted on to the sample pad. The LFS strips were placed into 2 ml Eppendorf tubes containing 80 µL of LFS Running Buffer [35] for 5 minutes. LFS were scanned using an Epson Perfection V39 Flatbed Scanner (Epson, New South Wales, AUS). The scanned images were converted to greyscale using Irfan View 64 and then imported to ImageJ for analysis. Band intensity analysis and statistical quantification were conducted as previously described [36].

### Sensitivity and Specificity testing

Analytical sensitivity testing for Iso-CHIKV-Dx assays was performed using a 10-fold serial dilution of purified, synthetic RNA transcripts coding for CHIKV E1 gene (LC500222.1). Analytical specificity testing utilised purified RNA of various virus strains (Table 2). Due to a large variation in viral load copy number in patients infected with CHIKV, a particular value for infection simulation could not be established. However, the benchmark detection limit for CHIKV diagnostic is approximately 20 copies numbers per reaction [37]. As such, synthetic CHIKV RNA transcript (1 x 10^4^ copies/µL) was used as a positive control to ensure validation of specificity and establish a reference point for sensitivity.

**Table 2:** Virus strains used in this study.

Virus	Abbreviation	Strain	GenBank accession number
Alphavirus chikungunya (Chikungunya virus)	CHIKV	ARUBA-15802650	LC500222.1
Alphavirus chikungunya (Chikungunya virus)	CHIKV	Mauritius	EU404186
Alphavirus Onyong (O’nyong nyong virus)	ONNV	IMTSSA/2004/5163	DQ383272
Alphavirus sindbis (Sindbis virus)	SINV	MRE-16	AF492770
Alphavirus semliki (Semliki forest virus)	SFV	Tanzania53	MK280688.1
Alphavirus rossriver (Ross River virus)	RRV	Australia/1972/14389	MK028845.2
Alphavirus barmahforest (Barmah Forest virus)	BFV	MIDIWBTA.2018	MN064697.1
Alphavirus mayaro (Mayaro virus)	MAYV	BeH407	QDL88200.1
Orthoflavivirus dengeui (Dengue virus) serotype 1	DENV-1	ET00.243	JN415499
Orthoflavivirus dengeui (Dengue virus) serotype 2	DENV-2	ET00.300	JN568254
Orthoflavivirus dengeui (Dengue virus) serotype 3	DENV-3	East Timor 2000	JN575585
Orthoflavivirus dengeui (Dengue virus) serotype 4	DENV-4	ET00.288	JN571853
Orthoflavivirus zikaense (Zika virus)	ZIKV	MR766	KX830960
Orthoflavivirus japonicum (Japanese encephalitis virus)	JEV	Nakayama	EF571853

### Mock-infected urine

CHIKV culture 2.5 x 10^7^ FFU/ml was spiked (1:10 dilution) into a tube containing artificial urine medium (Pickering Laboratories #1700–0018) commonly used for growing urological pathogens (Walker Scientific Pty Ltd, Joondalup DC, AUS). Final concentration of viral titre in simulated urine sample was 2.5 x 10^6^ FFU/ml. Rationale for 1:10 dilution was to reduce the titre to more realistic (real world) concentrations as the initial titre could be considered too high for a real-life simulation.

### Viruses and cell culture

All viral strains used in this study are listed in (Table 2).

### Virus culture

CHIKV ‘Mauritius strain’ culture was grown by infecting C6/36 cells at an M.O.I of 0.1 for 8 days. Culture supernatant was then clarified via centrifugation at 12,000 x g for 15 mins at 4°C. Clarified virus stock was then harvested and stored at -80°C.

### Cell culture

*Aedes albopictus* larvae cells C6/36 (ATCC-CRL-1660) were obtained from the ATCC. Culture of C6/36 cells was implemented as previously described [36].

### Titre determination

CHIKV titres were determined by immuno-plaque assay (IPA) using C6/36 cells in a 96 well plate as previously described [38]. Hybridoma supernatant of the 5.5G9 mouse monoclonal antibody (mAb) at a 1:100 dilution was used as the primary antibody. Primary antibody 5.5G9 targeted the nucleocapsid protein of CHIKV, as previously characterised [39].

### LOD of CHIKV (Mauritius strain) Immuno Plaque Assay

Limit of detection (LOD) was established by performing an Immuno plaque assay (IPA) as described previously [38], using serially diluted 2.5 x 10^7^ FFU/ml CHIKV virus culture. Plaques were then counted as described in [38]. The LOD for this CHIKV IPA assay resulted in 25 FFU/mL which equated to 1.39794 log_10_ FFU/mL.

### CHIKV inactivation testing

CHIKV inactivation testing was performed using CHIKV ‘Mauritius strain’ culture (2.5 x 10^7^ FFU/ml) treated with TNA-Cifer Reagent E (TCE; BioCifer, Auchenflower, AUS) and PBS as a control at two ratios (1:1 and 2:1, CHIKV virus culture to TCE or PBS). Rationale for ratios (1:1 and 1:2) were chosen to balance the requirement of complete inactivation of a risk groupe-3 (BSL-3) virus (CHIKV), whilst also limiting any degradation of viral genomic RNA from the toxicity of TNA-Cifer Reagent E. A series of three passages for each time point and respective ratio was titred to establish complete inactivation.

### RNA purification

RNA from viral isolates were purified using TRIzol (Invitrogen by Thermo Fisher Scientific Pty Ltd, Victoria, AUS). Virus isolate stocks (Table 2) were generously provided by the Hobson-Peters lab (University of Queensland) and Andreas Suhrbier lab (QIMR Berghofer Medical Research Institute). All virus isolates were exhibited titres of at least 1 x 10^6^ FFU/ml or greater as a benchmark measure to ensure equal and sufficient viral RNA. Viral RNA was eluted into 50 µL of nuclease free H_2_O and stored at -80°C.

### CHIKV qRT-PCR

CHIKV infected urine samples were placed in TRIzol (Life technologies, Carlsbad, CA, USA). RNA extraction and purification was then performed as described above. cDNA was then generated with Super script III First Strand Synthesis System (Invitrogen by Thermo Fisher Scientific Australia Pty Ltd, Victoria, AUS) according to the manufacturer’s instructions using 1 µg of purified RNA. Quantitative real-time PCR (qRT-PCR) was performed in a reaction consisting of 2µL of cDNA, 10 µL of 2X SYBR green (QuantiNova, Qiagen, Germany) PCR master mix, 6 µL of nuclease free H_2_O and 1 µL of 10 µM forward and reverse primers (FW: 5’-GACGTCTATGCTAAYACHCAACTGGTACTGC-3’; REV: 5’-CTCTTACCGGGTTTGTTGCTATTTGRCAGCC-3’) in a total reaction volume of 20 µL. Real time-RT-qPCR products were detected using Rotorgene 6000 (Corbett research, Mortlake, Australia) under the following cycling conditions: one cycle of 95°C for 2 min, 45 cycles of 94°C for 5 sec, 60°C for 10 sec and 72°C for 30 sec. A standard melt curve was run to confirm PCR products. Each sample was analysed in triplicates and normalised to RNA transcripts generated from the sensitivity test. Data were analysed using Rotor-Gene Real-Time Analysis software (Corbett, Mortlake, Australia).

Data availability: Balea, Rickyle; Amarilla, Alberto A; Hobson-Peters, Jody et al. (Forthcoming 2025). Development and proof of concept evaluation for a low resource compatible Chikungunya virus diagnostic [Dataset]. Dryad. https://doi.org/10.5061/dryad.547d7wmmf

## Results

### Analytical sensitivity and specificity

**Fig 2:**
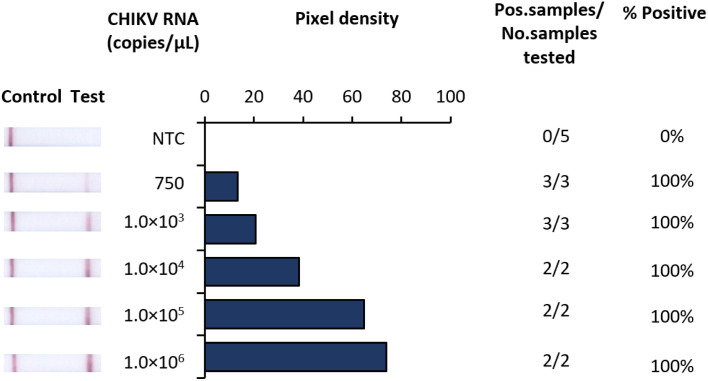
Analytical sensitivity of Iso-CHIKV-Dx using synthetic RNA transcripts. Assays were conducted against 10-fold dilutions of synthetic CHIKV RNA. The presence of two lines (control and test) on scanned images of the lateral flow strips is indicative of a positive result (far left). RNA concentration/µL and No template control (NTC) consisted of nuclease free H_2_O in place of RNA (left). Numeric analysis of normalised pixel density of bands contrasted with the white space (middle). Number of positive samples detected over number of samples tested along with calculated percentage accuracy of positive results for each dilution series (right).

In developing a rapid, low-resource compatible diagnostic, we strategically designed RT-RAA primers and probes to target a highly conserved region of the E1 gene present among all known CHIKV lineages. To evaluate the analytical sensitivity of our RT-RAA assays were tested against serial dilutions of purified CHIKV RNA transcripts. Under these conditions the limit of detection (LOD) of RT-RAA was found to be 750 RNA copies/µL of RNA transcripts (Fig 2). Due to the qualitative aspect of our diagnostic, pixel density was analysed using ‘Image J’ to establish a quantifiable value of detection. As such, the pixel density values analysed from scanned lateral flow strips provided an indicative numerical value of the approximate RNA copy numbers detected from the lateral flow strip read out.

**Fig 3:**
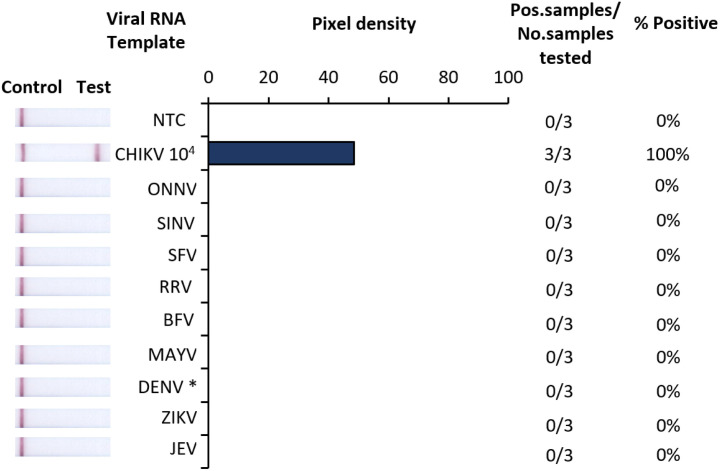
Analytical specificity of Iso-CHIKV-Dx using alphaviruses and co-circulating flaviviruses. Specificity assays used synthetic RNA from CHIKV (PTC) and TRIzol extracted RNA from O’nyong nyong virus (ONNV), Sindbis virus (SINV), Semliki Forest virus (SFV), Ross River virus (RRV), Barmah Forest virus (BFV), Mayaro virus (MAYV), Zika virus (ZIKV), Japanese encephalitis virus (JEV) and all 4 Dengue virus (DENV) serotypes. CHIKV 10^4^ copies/µL used as positive control; whilst No template control (NTC) consisted of nuclease free H_2_O in place of RNA (left). Numeric analysis of normalised pixel density of bands contrasted with the white space (middle). Number of positive samples detected over number of samples tested along with calculated percentage accuracy of positive results for each dilution series (right).

The analytical specificity of Iso-CHIKV-Dx was assessed using six common alphaviruses that share genomic similarity with CHIKV (Table 2). Additional assays were conducted using ZIKV, JEV and the 4 DENV serotypes as these virus’ commonly co-circulate in the same vectors and geographic location as CHIKV. Our results showed that Iso-CHIKV-Dx was negative for the common six alphaviruses and co-circulating flaviviruses (Fig 3).

### Sample preparation inactivates CHIKV

**Fig 4:**
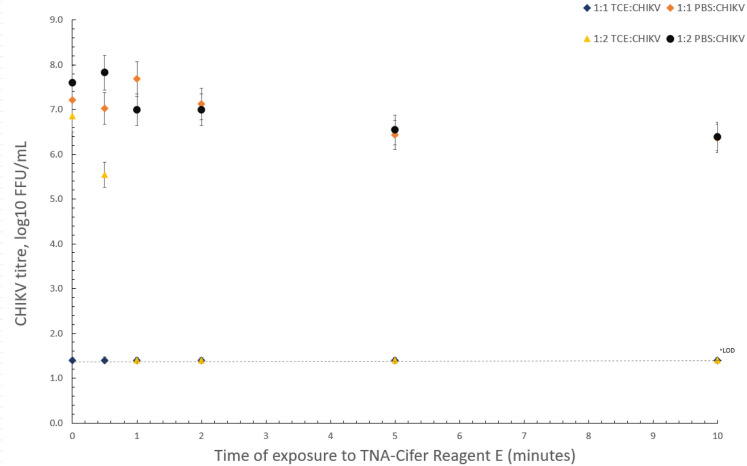
Inactivation of CHIKV (Mauritius strain) culture using TNA-Cifer Reagent E. Inactivation of 2.5 x 10^7^ FFU/mL CHIKV (Mauritius strain) culture by TNA-Cifer Reagent E (TCE). Samples were mixed in at 1:1 and 2:1 ratio (sample to TCE) and incubated between 0 and 10 minutes at room temperature. *The limit of detection (LOD) for CHIKV (Mauritius strain) via immuno plaque assay.

A rapid, low resource compatible diagnostic requires sample preparation methodology that is implementable in low resource settings with minimal to no prior training. Here we assessed the capacity of TNA-Cifer Reagent E (TCE) as a sample preparation reagent for rapid inactivation and sample preparation of CHIKV. Our results showed that CHIKV ‘Mauritius strain’ (2.5 x 10^7^ FFU/mL) was inactivated from immediate exposure of TCE time point (0 minutes) at a 1:1 (TCE:CHIKV) ratio and from 1 minute onwards at a 1:2 (TCE:CHIKV) ratio (Fig 4).

### Detection of CHIKV in synthetic urine

**Fig 5:**
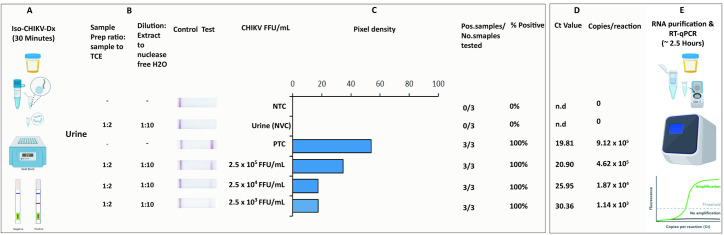
Rapid Iso-CHIKV-Dx of mock urine samples spiked with CHIKV (Mauritius). **A)** Workflow, equipment needed and time frame of Iso-CHIKV-Dx. **B)** Sample processing conditions including sample to reagent ratio and processed sample dilution ratio. **C)** Sample description and quantities (NTC, non-template control; PTC, positive template control, synthetic CHIKV RNA transcripts 10^6^ copies/µl); NVC, no virus control, urine. Scanned lateral flow strips showing test and control bands observable by naked eye. Normalised pixel densities (black values) from the displayed lateral flow strips. **D)** Comparative Ct values and copies/reaction quantified via TaqMan qRT-PCR. **E)** Workflow, equipment and time frame involved in conventional CHIKV RT-PCR diagnostic. (Images used to create this figure was obtained from Bio render-https://www.biorender.com).

Urine is a rapid, non-invasive sample collection option frequently used in arbovirus diagnostics [40,41]. To evaluate the effectiveness of Iso-CHIKV-Dx with urine we simulated an infectious clinical sample using synthetic urine spiked with CHIKV (Mauritius strain). Whilst complete inactivation was observed at both 1:1 and 1:2 ratios after 2 minutes, 1:2 for 5 minutes was chosen as the preferred ratio and time to ensure the higher RNA integrity and reduced likelihood of inhibitors. Using the same simulated urine clinical sample, we performed a comparative diagnostic consisting of our Iso-CHIKV-Dx and a qRT-PCR run in parallel. Our rapid, Iso-CHIKV-Dx demonstrated a LOD of 2.5 x 10^3^ FFU/mL of live CHIKV (Mauritius strain) (Fig 5). The quantified RT-qPCR equivalent was calculated to be a Ct value of 30.36 and 1,140 copies of CHIKV RNA/reaction. Due to the volume of template (2µL) used with the RT-qPCR, our equivalent LOD was calculated to be 570 copies/µL (1,140/2) of CHIKV RNA.

## Discussion

While a vaccine for CHIKV has been approved in the United States [10], there are still several countries and regions where CHIKV is endemic of which the approval has not been established. Combined with a lack of available medicines and therapeutics, CHIKV remains a significant health risk to individuals and populations in numerous regions throughout the world. As CHIKV infections are primarily observed in remote tropical locations that lack experienced health care workers and infrastructure, simple point-of-care diagnostic strategies such as iNAATs are likely to have more practical compatibility than conventional methods such as RT-qPCR.

In this study, we developed and evaluated a novel iNAAT (Iso-CHIKV-Dx) which combines a rapid, low resource compatible sample processing protocol with RT-RAA and lateral flow strip detection to achieve efficient detection of CHIKV in urine in under 30 minutes. We demonstrated that the analytical sensitivity of our Iso-CHIKV-Dx was 750 copies/µL using synthetic RNA generated from RNA transcripts of the E1 gene cloned into a bacterial plasmid. As various studies tend to use various metrics for limit of detection, the benchmark 20–200 copies/reaction previously described [37] was used to validate sensitivity. Given that our reaction consisted of 1 μL of sample template, the back calculation based on the referenced benchmark for isothermal approaches for CHIKV detection described [37] validated that our sensitivity was comparable. We also confirmed 100% specificity where our test did not detect any of the closely related *Alphaviruses* nor any of the common co-circulating *Flaviviruses*. This was a particularly crucial aspect due to the high genetic similarity CHIKV shares with several *Alphaviruses*, particularly O’nyong nyong virus (ONNV) [42]. Using live virus in artificial urine samples, we demonstrated that our Iso-CHIKV-Dx achieved a LOD of 2.5 x 10^3^ FFU/mL of CHIKV (Mauritius strain), which equates to 570 copies/µL of RNA and a Ct value of 30.36 via RT-qPCR.

TNA-Cifer Reagent-E has previously demonstrated effective inactivation of other pathogens such as ZIKV [43], Nipah virus (NIV) [29], Hendra virus (HeV) [44] and DENV [36], whilst ensuring sufficient extraction of viral RNA. Our data show that this reagent was also effective in inactivating CHIKV and extracting viable viral RNA for the iso-CHIKV-Dx assays. With a timeframe of only 30 minutes, compared to the 2.5 hours involved via RNA isolation and RT-qPCR, our Iso-CHIKV-Dx exhibits promising qualities for a rapid, point-of-care diagnostic, particularly when considering low resource settings. Our sensitivity of 570 RNA copies/µL from live CHIKV (Mauritius strain) is highly comparable to a 1-step real time RT-LAMP CHIKV assay that reported analytical sensitivity of 1,000 RNA copies/reaction [45]. A separate CHIKV RT-LAMP study did however, report sensitivity of 12 copies/reaction extracted from serum samples [46]. However, whilst optimised RT-LAMP assays are compatible with low resource settings, the assay does require multiple primers. Although these primers are not probe based, primer design for highly divergent RNA viruses have been known to be an issue with LAMP assays [47]. In contrast our Iso-CHIKV-Dx assays utilises a rapid, simple sample processing procedure, involves fewer steps (no RNA purification required) and generates a result in 30 minutes at a constant 39°C. Furthermore, RT-RAA, a key component of our Iso-CHIKV-Dx, has demonstrated high tolerance to PCR inhibitors [48]. In this regard our Iso-CHIKV-Dx test demonstrates many of the necessary characteristics to fulfill the R.E.A.S.S.U.R.E.D (Real-time connectivity, Ease of specimen collection, Affordability, Sensitivity, Specificity, User friendly aspect, Rapidity, Equipment reliability and how Deliverable to end users) criteria more comprehensively than RT-LAMP based assays [49].

Due to biosecurity and quarantine constraints, we were unable to assess our diagnostic against a wider panel of live CHIKV strains. Nevertheless, our extensive phylogenetic analysis provides strong evidence that our primers and probes anneal to all the known E1 gene targets present in all CHIKV strains. Moreover, the high conservation of the target across all lineages suggests that evolution of the E1 genes is unlikely.

RT-qPCR is likely to remain the preferred diagnostic technology in locations with advanced pathology laboratories and trained laboratory personnel. While urine can maintain viable CHIKV RNA for up to 95 days [50], blood, serum and semen are typically the most frequently used clinical matrices via conventional RT-qPCR diagnostics. The typical detection range for CHIKV in these clinical matrices via RT-qPCR is observed to be between 5–100 RNA copies per reaction [51–53]. Despite its high sensitivity, the vast majority of CHIKV outbreaks occur in rural, remote settings [54], RT-qPCR requires expensive thermal cyclers, extensive laboratory experience, costly reagents and strict storage requirements. Therefore, diagnostic approaches that utilise iNAAT’s are better suited for such low-resource environments.

While implementing a basic analogue reading device for ‘Real-time connectivity’ may be a minor obstacle, we are confident if required, a simple pocket scanner would suffice as a suitable real-time connectivity device. In the future we aim to evaluate the clinical efficacy of our Iso-CHIKV-Dx in field settings and validate its versatility by testing various CHIKV strains from different lineages. We aim to obtain live clinical samples for testing in mock laboratory settings or deploy our CHIKV test in regions experiencing sporadic CHIKV outbreaks.

Due to the manual steps required with our CHIKV test, it should be acknowledged that high throughput and large-scale screening of clinical samples would not occur as rapidly as with RT-qPCR. However, in contrast to RT-qPCR, our Iso-CHIKV-Dx was strategically designed to be field compatible, requiring minimal resources, no advanced instrumentation and no pathology expertise. Our data demonstrates that when compared to RT-qPCR (which typically takes 2.5 hours [55]), our diagnostic is approximately 4 times faster, with sample processing to result in a time frame of 30 minutes. While our test is not intended to replace RT-qPCR, our data suggests that it exhibits the necessary attributes to act as a potential pre-screening tool, particularly in early detection of CHIKV clusters in low resource settings. A potential obstacle our CHIKV test may encounter, as with many other POC-in field NAAT’s [56], is the risk of post-amplification cross-contamination. One potential solution to this issue could be substituting dTTP with dUTP, which has been shown to reduce the likelihood of cross-contamination in LAMP assays [57]. It should be noted that the feasibility of this measure has not been assessed with RAA. Alternatively, another approach could involve utilising a disposable cartridge [58] which has demonstrated effectiveness reducing post-amplification cross-contamination. Lastly, we acknowledge the limitation of samples tested within this study. Whilst the goal is to evaluate the performance of this Iso-CHIKV-Dx within a clinical setting, the novelty among all stages of the methodology resulted in more of a proof-of-concept validation. Future directions involving a pre-clinical evaluation of this study are currently being explored with real, patient samples.

Early detection of arboviruses such as CHIKV plays a vital role in mitigating potential outbreaks and preventing the spread of infections throughout communities. This is especially crucial in resource-limited areas, which often lack not only diagnostic tools but also treatment and clinical response capabilities. Thus, while it is beneficial to possess a suitable clinical detection tool for CHIKV infections, monitoring and surveillance should also be prioritized. Although unlikely to replace methods like RT-qPCR, Iso-CHIKV-Dx implemented in regions that are severely devoid of infrastructure, could serve as a potential first line indicator of infection that could assist in outbreak control. As mentioned previously, CHIKV is primarily vectored by two *Aedes aegypti* and *Aedes albopictus*. However, factors such as climate change and urban expansion have increased vector competence among arboviruses like CHIKV, making this an area of growing concern [59,60]. As such, a POC and field deployable assay that provides robust and rapid results would be considered highly beneficial for the surveillance and monitoring of CHIKV spread. Further investigations and evaluations of our Iso-CHIKV-Dx as a detection tool for infected mosquitoes and reservoir hosts are warranted. To that end, a study using TNA-Cifer Reagent-E combined with RT-RPA and lateral flow strips [61] demonstrated promising data, suggesting that our Iso-CHIKV-Dx could feasibly detect CHIKV-infected mosquitoes. Therefore, in addition to exploring clinical trials, we aim to evaluate the performance of our Iso-CHIKV-Dx as a tool for vector surveillance by testing mosquitoes as well [62].

## Conclusions

In conclusion, we developed and evaluated a pre-clinical CHIKV diagnostic platform that requires incubation at 39°C and produces a real-time diagnosis in under 30 minutes. Pre-clinical, proof-of-concept evaluation of our CHIKV test demonstrates promising innovations for novel detection and diagnostic methods for CHIKV. Our CHIKV test was able to detect down to 750 copies/µL of synthetic RNA and did not detect any non-specific *alphaviruses* or co-circulating *flaviviruses*. Using TNA-Cifer Reagent-E, we successfully inactivated live CHIKV (Mauritius strain) and extracted viable viral RNA at room temperature in 2 minutes. By combining our optimised sample processing protocol, RT-RAA and lateral flow strip detection, we developed a robust CHIKV detection platform. Compared to the ‘gold standard’ RT-qPCR, our data showed that our Iso-CHIKV-Dx has a LOD of 2.5 x 10^3^ FFU/mL of live CHIKV (Mauritius strain) equating to a Ct value of 30.36, and detection of 1140 copies of CHIKV RNA per reaction in urine samples. Due to the versatile and simplistic nature of our CHIKV test, we are confident that the practical applications of our Iso-CHIKV-Dx extend beyond clinical diagnostics. It has the potential to serve as a suitable tool for the surveillance and monitoring of vectors that transmit CHIKV and other arboviruses.
